# Improvement in Optoelectronic Properties of Bismuth Sulphide Thin Films by Chromium Incorporation at the Orthorhombic Crystal Lattice for Photovoltaic Applications

**DOI:** 10.3390/molecules27196419

**Published:** 2022-09-28

**Authors:** Tanzeela Fazal, Shahid Iqbal, Mazloom Shah, Bushra Ismail, Nusrat Shaheen, Hamad Alrbyawi, Murefah Mana Al-Anazy, Eslam B. Elkaeed, H. H. Somaily, Rami Adel Pashameah, Eman Alzahrani, Abd-ElAziem Farouk

**Affiliations:** 1 Department of Chemistry, Abbottabad University of Science and Technology (AUST), Abbottabad 22500, Pakistan; 2 Department of Chemistry, School of Natural Sciences (SNS), National University of Science and Technology (NUST), H-12, Islamabad 46000, Pakistan; 3 Department of Chemistry, COMSATS University Islamabad (CUI), Abbottabad Campus, Islamabad 22060, Pakistan; 4 Pharmaceutics and Pharmaceutical Technology Department, College of Pharmacy, Taibah University, Medina 42353, Saudi Arabia; 5 Department of Chemistry, College of Science, Princess Nourah bint Abdulrahman University, P.O. Box 84428, Riyadh 11671, Saudi Arabia; 6 Department of Pharmaceutical Sciences, College of Pharmacy, AlMaarefa University, Riyadh 13713, Saudi Arabia; 7 Research Center for Advanced Materials Science (RCAMS), King Khalid University, P.O. Box 9004, Abha 61413, Saudi Arabia; 8 Department of Physics, Faculty of Science, King Khalid University, P.O. Box 9004, Abha 61413, Saudi Arabia; 9 Department of Chemistry, Faculty of Applied Science, Umm Al-Qura University, Makkah 24230, Saudi Arabia; 10 Department of Chemistry, College of Science, Taif University, P.O. Box 11099, Taif 21944, Saudi Arabia; 11 Department of Biotechnology College of Science, Taif University, P.O. Box 11099, Taif 21944, Saudi Arabia

**Keywords:** chromium-doped, photovoltaic, thin film, solar harvesting, lattice parameters

## Abstract

By using the chemical bath deposition approach, binary bismuth sulphides (Bi_2_S_3_) and chromium-doped ternary bismuth sulphides (Bi_2−x_Cr_x_S_3_) thin films were effectively produced, and their potential for photovoltaic applications was examined. Structural elucidation revealed that Bi_2_S_3_ deposited by this simple and cost-effective method retained its orthorhombic crystal lattice by doping up to 3 at.%. The morphological analysis confirmed the crack-free deposition, hence making them suitable for solar cell applications. Optical analysis showed that deposited thin films have a bandgap in the range of 1.30 to 1.17 eV, values of refractive index (*n*) from 2.9 to 1.3, and an extinction coefficient (*k*) from 1.03 to 0.3. From the Hall measurements, it followed that the dominant carriers in all doped and undoped samples are electrons, and the carrier density in doped samples is almost two orders of magnitude larger than in Bi_2_S_3_. Hence, this suggests that doping is an effective tool to improve the optoelectronic behavior of Bi_2_S_3_ thin films by engineering the compositional, structural, and morphological properties.

## 1. Introduction

To satisfy the need for renewable energy, new efforts are required to efficiently gather incident photons [1,2,3]. First-generation photovoltaic devices, such as single-crystal silicon-based devices, although having an efficiency of up to 15%, are expensive to manufacture and install. While second-generation devices, i.e., polycrystalline semiconductor thin film-based solar cells, are cost-effective, their poor efficiency limits their applicability [4,5,6]. In the quest to design efficient, inexpensive solar cell absorbers, dye-sensitized solar cells [7] and lead halide perovskite, an emerging material with an efficiency ∼30%, have paved the way for the design of newer materials [8,9]. However, there are two main concerns for the mass production of conventional perovskite. First is the issue of the intrinsic chemical instability of lead halide perovskites and second is their toxic constituent, i.e., Pb [10]. To address these issues, lead-free and non-halide perovskites with earth-abundant constituency can be an option, although, as yet, no such candidate has demonstrated comparable performance [3]. The capture and transmission of charge carriers inside semiconductor networks have always been fundamental problems that have to be addressed in order to ensure efficient charge separation. To increase the overall effectiveness of light energy conversion, several strategies to enhance photoinduced charge separation and electron transfer processes have been put forward [11,12,13]. Additionally, it is a popular issue right now to concentrate future research efforts on the creation and use of unique nanostructures for the advancement of next-generation solar cells [14,15,16].

Metal chalcogenides are a kind of solar energy material with good optical and electrical characteristics that may be used in photovoltaic applications [5,17]. A number of binary and ternary chalcogenide semiconductor materials, such as CdSe, CdS_1−x_Se_x_, CdS, ZnSe, ZnS, Zn_1−x_Cd_x_S, Cd_1−x_Zn_X_S, Sb_2_S_3_, Sb_2_Se_3_, Bi_2_Se_3_, and Bi_2_S_3_, have been employed as semiconductor electrodes in solar cells [14,18,19,20]. Bi_2_S_3_ (bismuth sulphide) is a semiconductor material that belongs to the V-VI family. It is known as ‘bismuth glance’ or ‘bismuthinite’ when it occurs naturally in a grey crystalline form. Bi_2_S_3_ is a potential semiconducting material for optoelectronic appliances, with a band gap energy of 1.2–1.7 eV, making it suitable in thermoelectric and optoelectronic devices with a decent incoming photon to electron conversion efficiency (5%) [21]. Bi_2_S_3_ may be made using a variety of techniques, including electrodeposition, vapor deposition, sputtering, a solution gas interface, spray, and a chemical bath [22,23,24] in both non-aqueous and aqueous media. For the effective, straightforward, and easy deposition of large surface area thin films, the chemical bath deposition approach has been used [25]. In the chemical bath deposition method, both metal ions and chalcogen ions are released in a single bath and are then precipitated onto a film to form metal chalcogenides [26,27,28].

D block metals have high stability when used as dopants, decreasing semiconductor materials’ photo-corrosion restrictions. By causing reticular distortions in the semiconductor lattice, transition metals increase the percentage of faults, resulting in improved electron hole charge separation efficiency [29,30,31,32]. The dopant concentration and distribution, as well as the electron configuration and metal ion-electron donor density, all play roles in deciding the fate of the designed semiconductors [33,34,35]. Chromium is a lustrous, steely grey, hard, and brittle transition metal with the atomic number 24, which belongs to group 6. The strong corrosion resistance and hardness of chromium make it a valuable metal. In the current study, the inclusion of earth-abundant compatible Cr^3+^ ions into the Bi site of the orthorhombic crystal lattice is attempted in order to alter and enhance the characteristic behavior of Bi_2_S_3_.

The goal of this study is to evaluate the efficacy of the chemical bath deposition method to deposit chromium-doped Bi_2_S_3_ thin films for solar harvesting. Since impurity-induced chemical modification in semiconductor networks creates a favorable environment for the optoelectronic response, in the current study, we attempt to enhance optoelectronic properties of Bi_2_S_3_ thin films via structural and morphological manipulation with the help of a trivalent cation, i.e., Cr^3+^.

## 2. Results and Discussion

**Figure 1:**
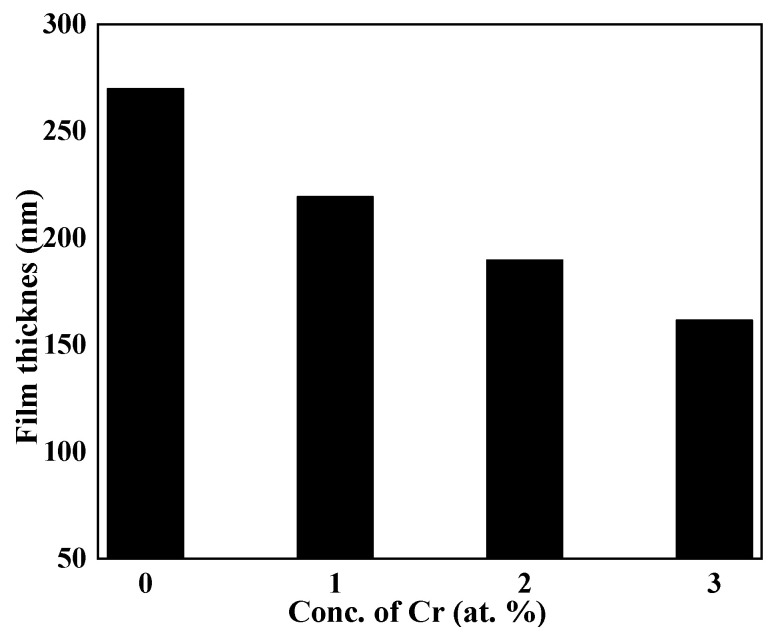
Variation in thickness versus concentration of dopant of undoped and Cr-doped Bi_2_S_3_thin films.

Figure 1 illustrates how an ellipsometeric method was used to gauge the films’ thickness. Film formation often slows as time goes on as a result of reactant consumption in reactions that typically start off quickly [32]. It is possible that the precipitation process was altered based on the thickness of the films with various Cr concentrations for the same deposition duration. The selectivity of EDTA for one metal ion over another and the ensuing difference in the strength of one metal-EDTA complex over another, i.e., Bi and Cr, are attributed to variations in the precipitation process and, finally, the film thickness [36]. Figure 1 reveals that Cr addition slowed down the precipitation reaction by strong chelation, developed between the Cr-EDTA [37,38,39], which resulted in a slow precipitation process by slowly releasing the Cr ions for the doped samples for the same period of deposition time, i.e., six hours.

**Figure 2:**
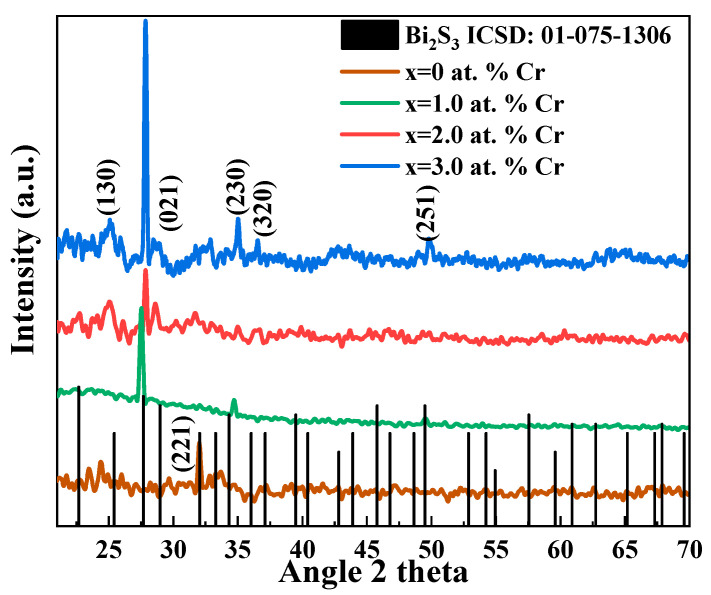
Bi_2_S_3_ thin films that have been Cr-doped and left undoped show various XRD patterns.

The XRD patterns are shown in Figure 2. The polycrystalline structure of the deposited thin films is evident by sharp and well-defined peaks. XRD analysis shows that both undoped and doped materials (Bi_2_S_3_ and Bi_2−x_Cr_x_S_3_) fit the bismuthinite phase of bismuth sulphide, with an orthorhombic structure (ICSD No: 01-075-1306), as shown by black vertical lines in Figure 2. The lack of additional peaks matching Cr or Cr-related, as well as Bi-related, peaks, suggests the formation of a single phase of Bi_2_S_3_ with high Cr homogeneity. For doped samples, a preferred orientation along the 021 plane is observed. Upon doping, the thin film growth process is influenced, resulting in the shifting of preferred planes [40].

Defects that are introduced as a result of dopant inclusion cause lattice deformation and shifts in the XRD peaks. The XRD peak locations move to either a higher or lower angle as a function of the external entity, i.e., dopant [41]. Change in the preferred orientation of thin films while transforming into the doped ones, as in the current case instead of the (221) plane to (021), is a common phenomenon [40]. Slide shifting of diffracted peaks at 2θ~35.0° and 49.0° towards larger angles give a clear indication of the incorporation of Cr ions in the Bi_2_S_3_ lattice [42]. Grain sizes were found to be decreased with the addition of Cr ions, as with the addition of Cr, more nucleation centers and sites are created for crystal growth. As both cations, i.e., Bi and Cr, act as seed nuclei, with the incorporation and increasing concentration of Cr ions, nucleation cites increased, resulting in a greater number of grains with a consequent reduction in size [43].

Lattice factors “*a*, *b*, and *c*”, unit cell volume “*V_cell_*”, Scherrer crystallite size “*D*” [44], X-ray density “*ρ_X-ray_*” dislocation density “ẟ”, and microstrain “Ɛ” were calculated using Equations (1)–(6).

(1) $$\frac{1}{d^{2}}⇄\;=\;\frac{h^{2}}{a^{2}}\;+\;\frac{k^{2}}{b^{2}}\;+\;\frac{l^{2}}{c^{2}}$$

(2) $$V_{cell}\;=\;abc$$

(3) $$D=\frac{k\lambda }{\beta \cos\theta_{B}}$$

(4) $$\rho_{X}{}_{\text{-}ray}=\frac{ZM}{V_{cell}N_{A}}$$

(5) $$ẟ=\frac{1}{D^{2}}$$

(6) $$Ɛ=\frac{4}{\tan\theta }$$

where *β* is the full width at half maximum intensity, *λ* is the X-ray wavelength and is equal to 0.15406 nm, *θ* is Bragg’s angle, *k* is the constant equal to 0.94, *Z* is the number of molecules per formula unit, and *M* is the molar mass. *V_cell_* and *N_A_* have their usual meanings. Crystallographic parameters calculated for both Bi_2_S_3_ and Bi_2−x_Cr_x_S_3_ thin films calculated from XRD data are tabulated in Table 1, which seem to be influenced by Cr addition. Transitions from binary to ternary, elemental to compound, and complex compounds often result in compositional and positional chaos [45].

**Figure 3:**
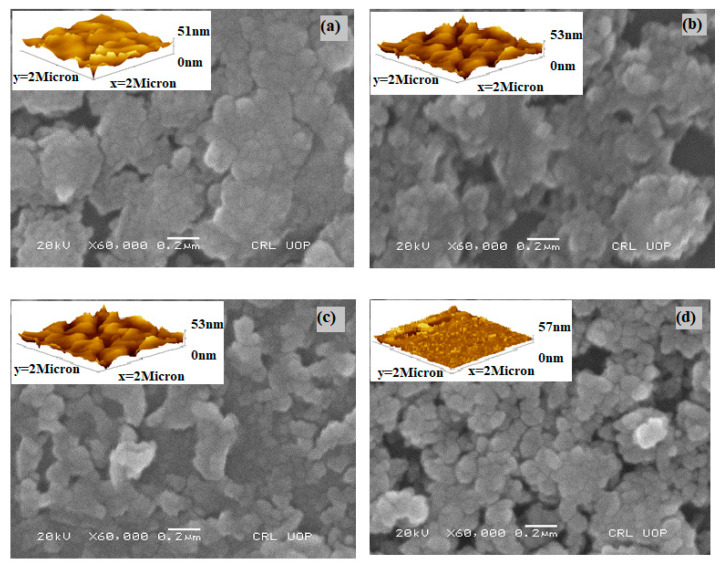
Morphological analysis of (**a**) 1 at.% Cr, (**b**) 1 at.% Cr, (**c**) 2 at.% Cr, and (**d**) 3 at.% Cr doped Bi_2_S_3_ with the help of AFM and SEM micrographs.

The surface morphologies of undoped Bi_2_S_3_ and Cr-doped Bi_2_S_3_ thin films are shown in Figure 3. A noticeable difference was observed between the morphological properties of films with the addition of Cr from 0–3 at.%. Figure 3a depicts the surface morphology of an undoped sample, which has compact, homogenous, and interconnected particles; Figure 3b depicts the surface morphology of a sample with 1% Cr, which has incredibly small particles that are comparable to those of pure Bi_2_S_3_, while the texture of the particles was preserved after Cr insertion. Upon further increase in the dopant, Figure 3c,d indicates irregular-shaped particles with a broad range of sizes. The particle size is in the range of 150 to 80 nm for all the deposited samples, which is in agreement with the XRD findings. Upon increasing the dopant concentration, the particle size decreased. As both cations, i.e., Bi and Cr, serve as seed nuclei for the growth of nanoparticles by Ostwald’s ripening, particles were discovered to grow at the cost of previously deposited particles, resulting in agglomeration owing to the overgrowth of microscopic grains on previously deposited particles with uneven boundaries. Higher dopant concentrations resulted in loosely organized, smaller-particle-sized films on the substrate as evident by both SEM and AFM studies, hence validating the findings of XRD data. Atomic force microscopic studies (inset figures) showed that with an increasing doping concentration, the thickness increased from 51 to 57 nm by offering more surface area for photon interactions. The differences in the compositions of the deposited samples, which are determined by the Cr-to-Bi ratio, are connected to variations in their morphologies at various dopant concentrations.

**Figure 4:**
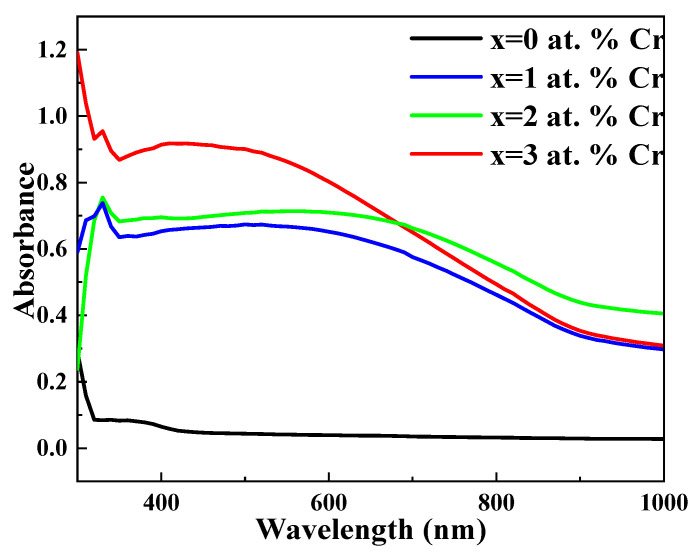
Absorbance spectrum of undoped and Cr-doped Bi_2_S_3_ thin films.

The absorbance-versus-wavelength plot of chromium-doped bismuth sulphide thin film systems is shown in Figure 4. The strong absorbance region in this figure is between 400 and 800 nm, while in the infrared region, there is noteworthy absorbance. Furthermore, the absorption in the near-infrared region harvests more photons to invert into photocurrent [46]. The position of the absorption edge shifts red as the Cr content increases from 0 to 3 at. Percent. By modifying the ratio of Bi and S atoms, the addition of Cr to the system changes the average atomization energy, leading to this shift. The red shift in the absorption spectra will be helpful to enhance the ability of the synthesized materials to absorb a wider spectrum of light (more in the visible region). Additionally, Table 2 shows the compositions’ optical absorption coefficients, which ranged from 10^5^ to 10^6^ cm^−1^, and confirms their potential as effective absorber materials for solar applications.

**Figure 5:**
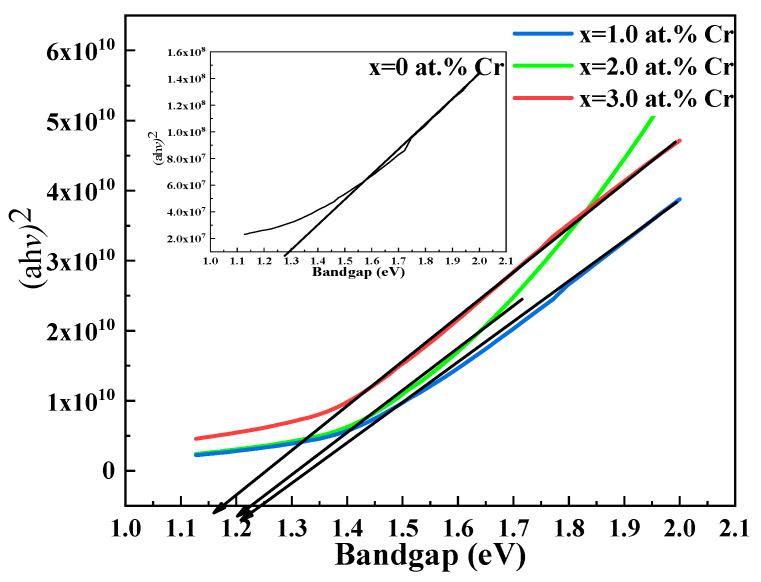
Tauc plot for undoped and Cr-doped Bi_2_S_3_ thin films.

Figure 5 illustrates the band gaps of the thin films, which were calculated using UV-Vis spectroscopy and the Tauc equation. These band gaps are in good agreement with known values and are appropriate for applications as visible light absorber materials [47]; the value of exponent *n* is 2, indicating a direct and allowed transition.

To calculate the band gap (*E_g_*) of deposited films, the *Tauc* equation is used:

(7) $${\left(\alpha h\nu \right)}^{r}\;=\;A(h\nu -E_{g})$$

“*h*” stands for Planck’s constant (6.62 × 10^−34^ Js), “*υ*” stands for light frequency, *A* is the constant, and “α” stands for the absorption coefficient calculated from this relationship.

**Figure 6:**
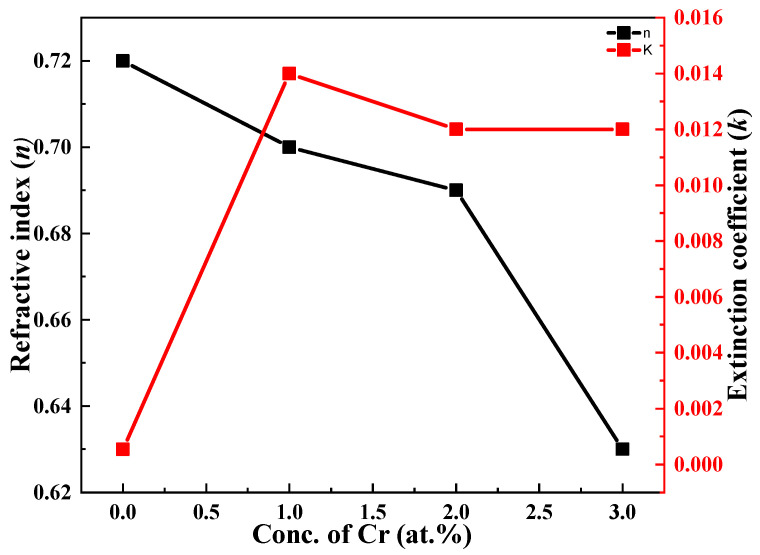
Dependence of refractive index and extinction for undoped and Cr-doped Bi_2_S_3_ thin films.

Regarding the dependence of the composition of thin films on the band gap, a decrease in the band gap as shown in Figure 6 is credited to manifestation in the band structure by introducing discrete impurity levels [48]. Crystallinity, an important factor, might also speculate its role in the decrement in the band gap [49].

Transmittance (T) and absorbance (A) are inter-related through the following equation:

A = −log (T)(8)

The absorption coefficient (*α*) is calculated by the following equation:

(9) $$\alpha (cm^{-1})\;=\;\frac{1}{d}\ln\frac{{\left(1-R\right)}^{2}}{T}$$

The value of the extinction coefficient is calculated using the absorption coefficient (*α*) and optical wavelength (*λ_o_*).

(10) $$k=\alpha \lambda_{o}/4\pi$$

Equation (11) demonstrates how to use the reflectance (*R*) and extinction coefficient (*k*) data to obtain the refractive index (*n*).

(11) $$n\;=\left(\frac{1+R}{1-R}\right)\;+\sqrt{\left(\frac{4R}{{\left(1-R\right)}^{2}}-k^{2}\right)}$$

The refractive index (*n*) and extinction coefficient are related to the dielectric constant (*k*).

(12) ε = (n − ik) 

The dielectric constant is connected to certain substances, such as those used in capacitors, printed circuit board substrates, and cable insulation. It is a complex number, with the imaginary component corresponding to dielectric losses and the real part (ε_r_) indicating the degree of the polarizability of a material. They were calculated by the relations:

(13) $$\varepsilon_{r}=\left(n2-k\right)$$

(14) $$\varepsilon_{i}=\left(2n-k\right)$$

Electrical conductivity (*σ_e_*) is estimated from the values of the wavelength (*λ*), refractive index (*n*), and speed of light (c = 2.8 × 10^8^ m/s). Equation (15) may be used to determine it mathematically.

(15) $$\sigma_{e}\;(\Omega {\mathrm{cm}}^{-1})=2\pi /\lambda \mathrm{nc}\;$$

Thermal conductivity (*σ_t_*) is assessed by Equation (16).

(16) $$\sigma_{t}\;(W/\mathrm{mK})=\mathrm{LT}\;\sigma_{e}\;$$

L is the Lorentz number, 2.45 × 10^−8^ W Ω K^−2^ and T is the temperature.

Table 2 shows absorption coefficient values that are suitable for use as an absorber layer in photovoltaic applications [50]. The real portion (ε_r_) of the complex dielectric constant describes how much light is retarded in the material, while the imaginary part (ε_i_) describes how much energy is absorbed from an electric field owing to the dipole signal. The real component of the dielectric constant is bigger than the imaginary part in this case, suggesting that the material’s reaction to light is visible and distinct [51]. Hence, the dielectric properties (ε) of materials contribute to mainly dipolar or orientation polarization, which arises from molecules that change their dipolar orientation when an electric field is applied. Values of both real and imaginary dielectric constants lie in the visible region, and this behavior leads to increased electronic transfers through the material from the valence band to the conduction band [50]. The Urbach energy, another critical optical characteristic of the material, is related to the width of the band tail of the localized states in the bandgap (E_u_^o^). The Urbach energy value is determined by the degree of defect in the chalcogenides [52]. The Urbach slope is calculated by plotting the logarithm of alpha versus photon energy and fitting a line after determining the linear zone. The width of tail states into the forbidden gap is quantified by E_u_^o^, which is the inverse of that slope. Table 2 shows the Eu values determined for undoped and doped thin films. The value of E_u_^o^ is zero in a perfect semiconductor. The Urbach energy was found to increase from 0.26 to 0.35 eV when the bandgap region below the bandgap became wider and included more tail-absorbing states. E_u_^o^’s behavior is shown to be influenced by Cr content, since Cr incorporation resulted in an increase in the number of abnormalities and diseases.

Figure 6 shows the considerable compositional dependence of doping and doping concentration on the refractive index and extinction coefficient. Values of the refractive index (*n*) decreased from 0.72 to 0.62, while an enhancement of the values of the extinction coefficient (*k*) was observed, i.e., from 0.001 to 0.012.

**Figure 7:**
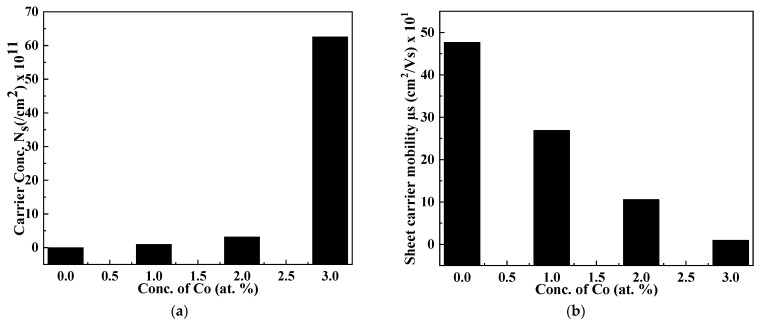
Dependence of (**a**) carrier concentration and (**b**) sheet carrier mobility on dopant concentration.

The incorporation of the Cr dopant strongly influenced the resistivity and conductivity of thin films, as shown in Figure 7a,b. The conductivity of binary thin films was enhanced up to 1.54 × 10^−2^ ohm^−1^ cm^−1^, and a consequent decrease in the resistivity of films from 299.9 to 59.0 Ω cm upon transforming the binary compound to a ternary compound is observed (Table 3). The value of sheet carrier mobility (*μ*_s_) is calculated and found to be 47.7 cm^2^/V s, which is considered to be higher than those published before in the literature (i.e., 28 cm^2^/V s) [53]. The value of carrier concentrations (*N*_s_) decreases while *μ*_s_ increases with an increasing concentration of Cr, as shown in Figure 7. Recombination of the stimulated carriers by the traps, which may be a shadow or deep, led to a decrease in mobility [54]. The behavior of thin films in the present study is n-type.

**Figure 8:**
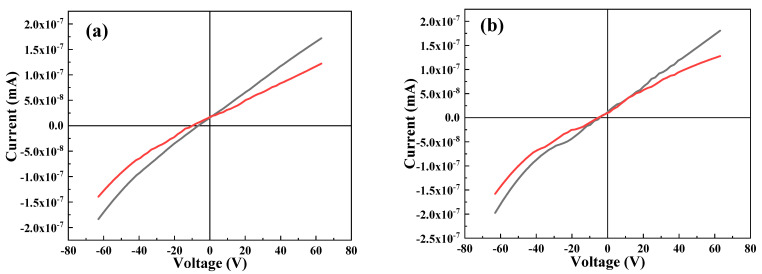

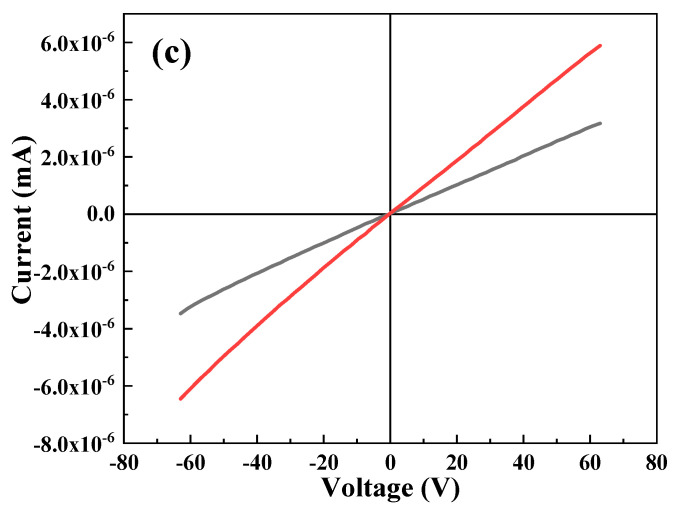
IV study of (**a**) 0 at.% Cr, (**b**) 1. 0 at.% Cr, and (**c**) 3.0 at.% Cr.

Figure 8 depicts the IV behavior of undoped and selected Cr-doped thin films. It is clear that with an increasing Cr content, the diode behavior of the film is enhanced, hence making the ternary material more suitable for photovoltaic applications. An improvement in the photocurrent signal of treated Bi_2_S_3_ compared to that of pure Bi_2_S_3_ under visible-light irradiation has also been reported previously [55].

## 3. Materials and Methods

Well-cleaned commercially available soda-lime microscopic glass slides were used as substrates. The chemicals used were Bi(NO_3_)_3_·5H_2_O, chromium nitrate pentahydrate (Cr(NO_3_)_3_·9H_2_O, Sigma, Schnelldorf, Germany, 98%), thioacetamide (CH_3_CSNH_2,_ Aldrich, 99%), nitric acid (HNO_3_, Sigma), and ethylene-diamine-tetra-acetic-acid, (EDTA, Sigma, 99%). To deposit pure and chromium-doped samples in the range of 1–3 at%, four baths with varying concentrations of Cr(NO_3_)_3_·9H_2_O and Bi(NO_3_)_3_·5H_2_O were prepared to deposit undoped and doped films, labeled as 0 at.% Cr, 1.0 at.% Cr, 2.0 at.% Cr, and 3.0 at.% Cr. Equal-volume and equimolar (10 mL of 0.10 M) bismuth nitrate and EDTA solutions for the pure sample were mixed in a bath at pH 2. To synthesize the doped derivative samples, different concentrations of the chromium solution were added to the same bath. Thioacetamide (10 mL of 0.1 M) was added to the resultant mixture as the sulphur source. Pre-cleaned-glass films were placed vertically in the resultant mixture beaker for six hours at room temperature.

In order to assess how the planned material will behave, synthetic samples were exposed to various characterizations. Using a PANalytical Xpert’ Pro (Holland) X-ray Diffractometer, the phase composition of the deposited thin films was investigated using an X-ray diffraction study in the 20–700 range with Cu K irradiation (k = 0.15406 nm). Optical analysis was performed using the Perkin Elmer Lambda 25 spectrophotometer. Investigation of the morphology and content of samples was carried out using the JSM-6360A SEM and the ‘Contact mode AFM’ nasoscope digital equipment with a silicon nitride cantilever. Using a nano-chip dependability grade Hall effect device, the Hall experiments were examined. The optical properties of thin films were verified using the Systronics-117 spectrophotometer’s ellipsometry method (sensor). Additionally, the Keithley-2635A source meter was used to assess IV behavior while in ohmic contact with an Ag electrode.

## 4. Conclusions

Low bandgap energy, preferably in the visible range, high surface area, and conductivity are prerequisite properties for an efficient photocatalytic material. In the current study, chromium-doped bismuth sulphide thin films with good lateral homogeneity and an energy bandgap between 1.3 and 1.15 eV were successfully deposited in an acidic medium via the chemical bath deposition technique. The optical characteristics of the films were modified by dopant incorporation by modifying the lattice parameters and thickness of the films, according to a correlation between the optical band gap and lattice parameters of the films. According to the films’ optical properties, almost all of them were found to be efficient absorbers in the targeted UV-Vis range. Top-view scans and AFM observations indicate that the surfaces of the films were affected by the Cr contributions. We determined that the Cr concentration in the ternary chromium-doped bismuth sulphide chalcogenide had an effect on all of the distinctive characteristics of the deposited films without disrupting the crystal lattice. It is necessary to relate the influence of the dopant concentration on the distinctive characteristics at the same thickness by altering the deposition duration, since all optoelectronic properties rely on the thickness of the film.

## Figures and Tables

**Table 1:** Crystallographic parameters calculated from XRD data for thin films.

Cr Conc.	Calculated Lattice Constant				Average Crystallite Size (nm)	X-ray Density (gcm^−3^)	Dislocation Density cm^−2^	Average Microstrain × 10^−3^
	a (Å) 11.11 *	b (Å) 11.25 *	c (Å) 3.97 *	Volume of Cell (10^6^ pm^3^) 496.42 *				
x = 0	11.11	11.71	3.52	457	141		7.4	2.1
x = 1	11.12	11.22	4.16	519	72	8.48	0.65	1.4
x = 2	11.06	10.73	4.03	502	54	3.39	0.68	2.8
x = 3	11.08	11.28	3.94	492	72	19.1	0.69	2.1

* Standard values for the ICSD (Bi_2_S_3_) 01-075-1306.

**Table 2:** Optical parameters of selected samples calculated by UV-vis spectroscopy at 535 nm.

Parameters	Conc. Of Cr (at.%)			
	0	1.0	2.0	3.0
α × 10^4^ (cm^−1^)	80.90	333.9	315.59	319.30
€_i_	0.0008	0.0260	0.0238	0.0230
€_r_	0.5290	0.4896	0.4810	0.4828
€	0.5282	0.4638	0.00045	0.4595
σ_e_ × 10 (Ω cm)^−1^	0.293	1.75	0.4517	1.64
σ_o_ × 10^15^ (s^−1^)	1.33	6.23	5.93	4.88
σ_t_ × 10^−4^ (Ω cm/K)	20	4	1.64	6.0
E_u_^o^	0.26	0.33	0.34	0.35

**Table 3:** Hall studies of undoped and Cr-doped Bi_2_S_3_ films.

Cr Conc (at.%)	I (µA)	Resistivity Rho (ohm cm) × 10^1^	Conductivity Con (1/ohm cm) × 10^−2^	Carrier Concentration Ns (/cm^2^) × 10^11^	Sheet Carrier Mobility µs (cm^2^/Vs) × 10^1^
0	0.1	29.9	0.00393	0.016	47.7
1	0.1	21.2	2.14	0.950	26.9
2	0.1	6.48	2.12	3.19	10.6
3	0.1	5.90	1.54	62.6	1.01

## Data Availability

The data will be available on request.
